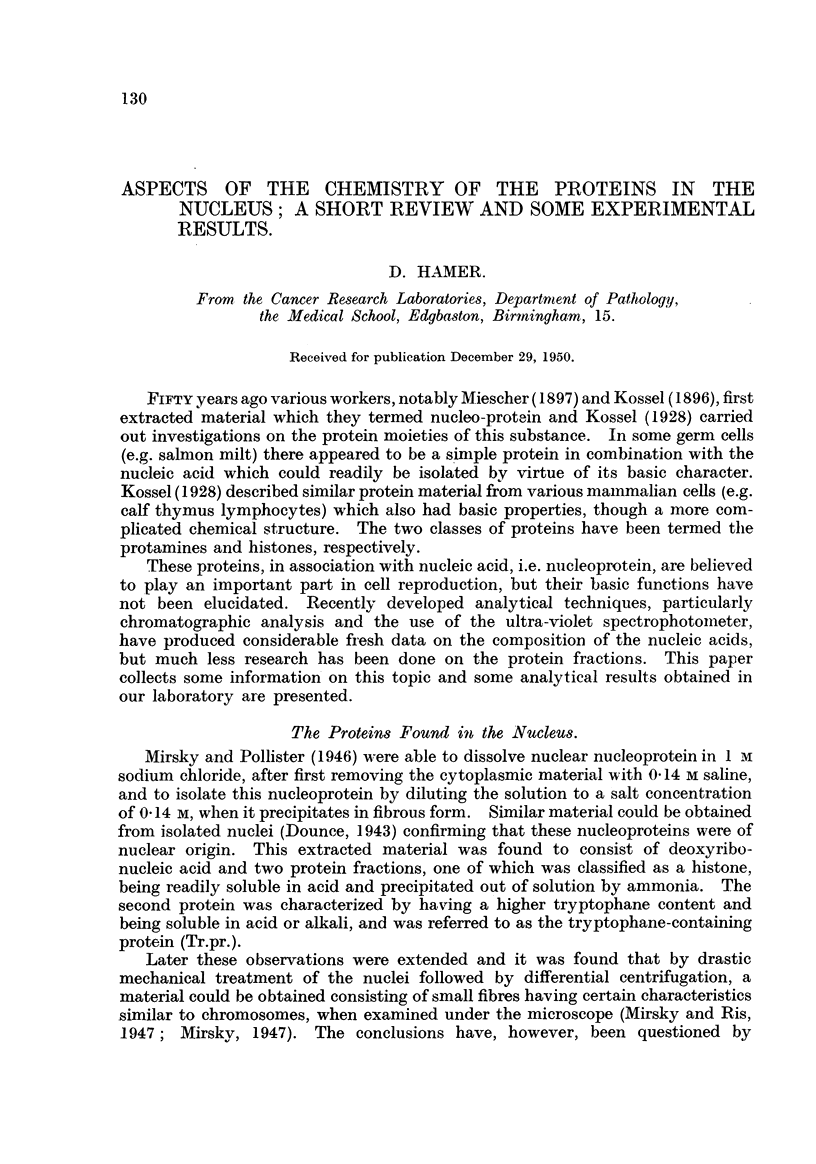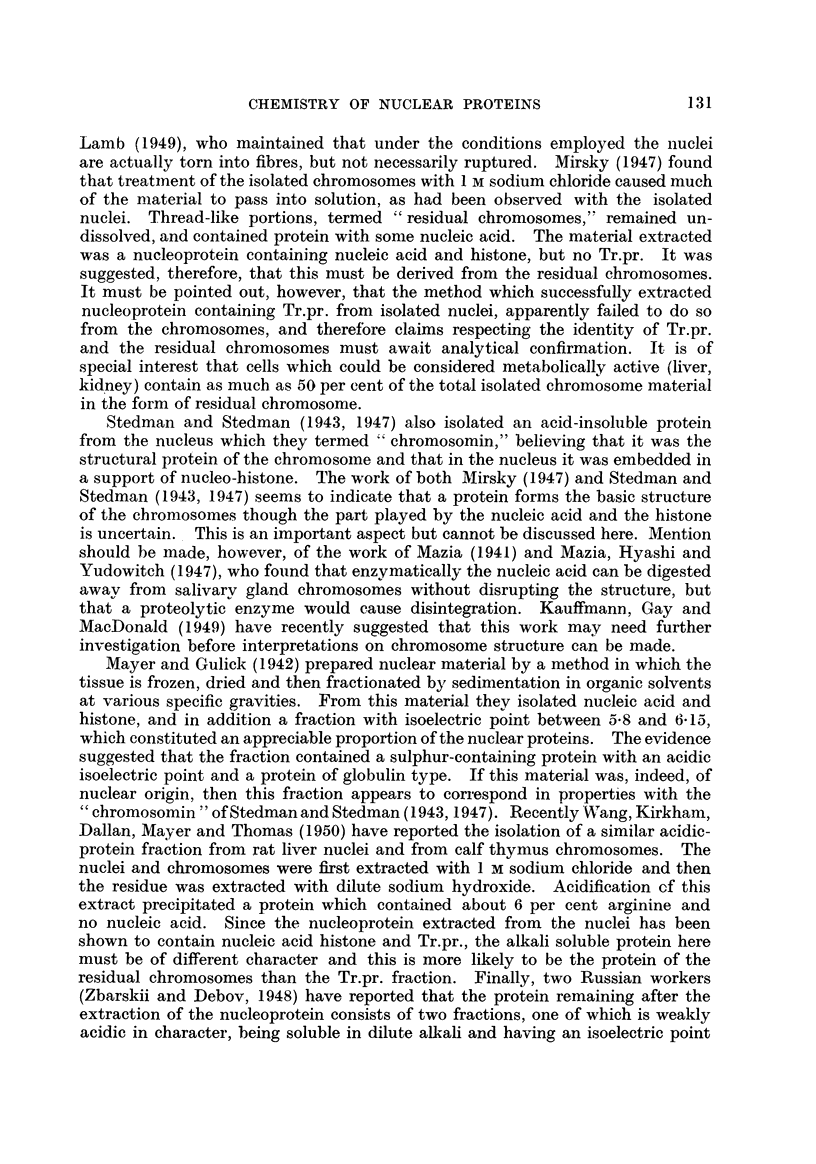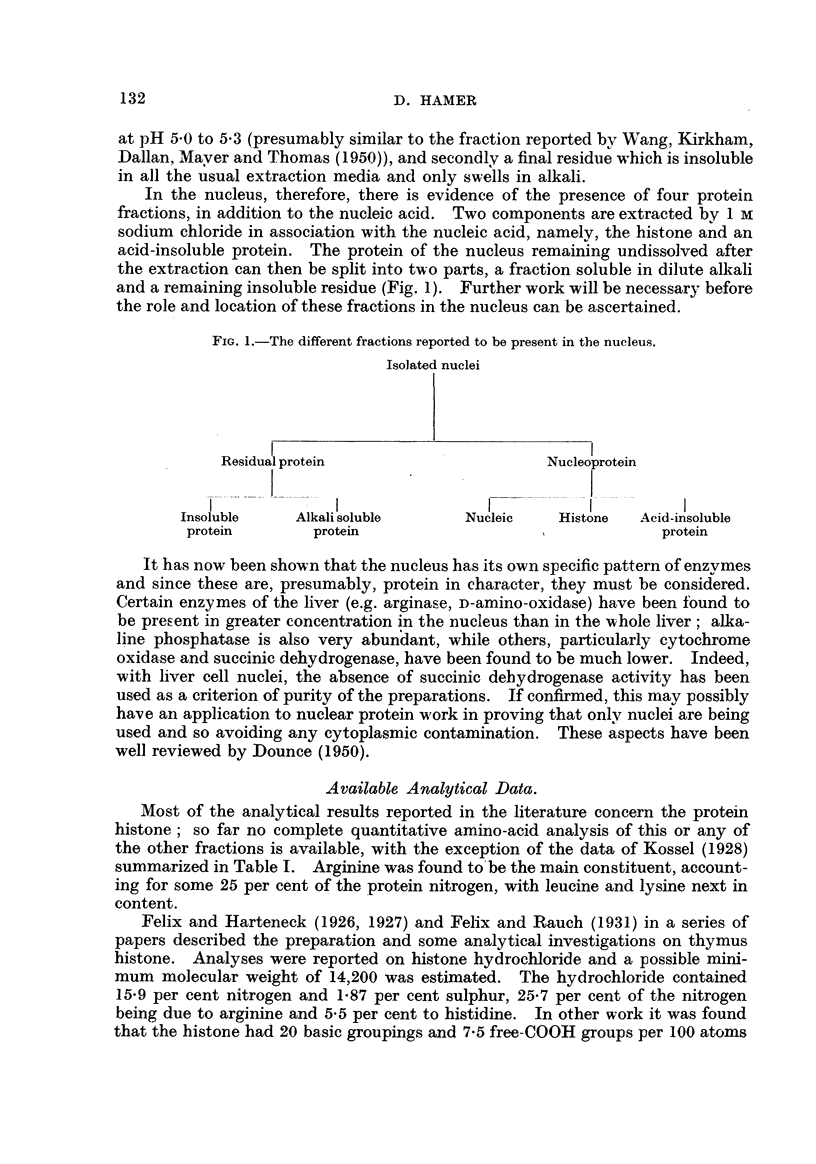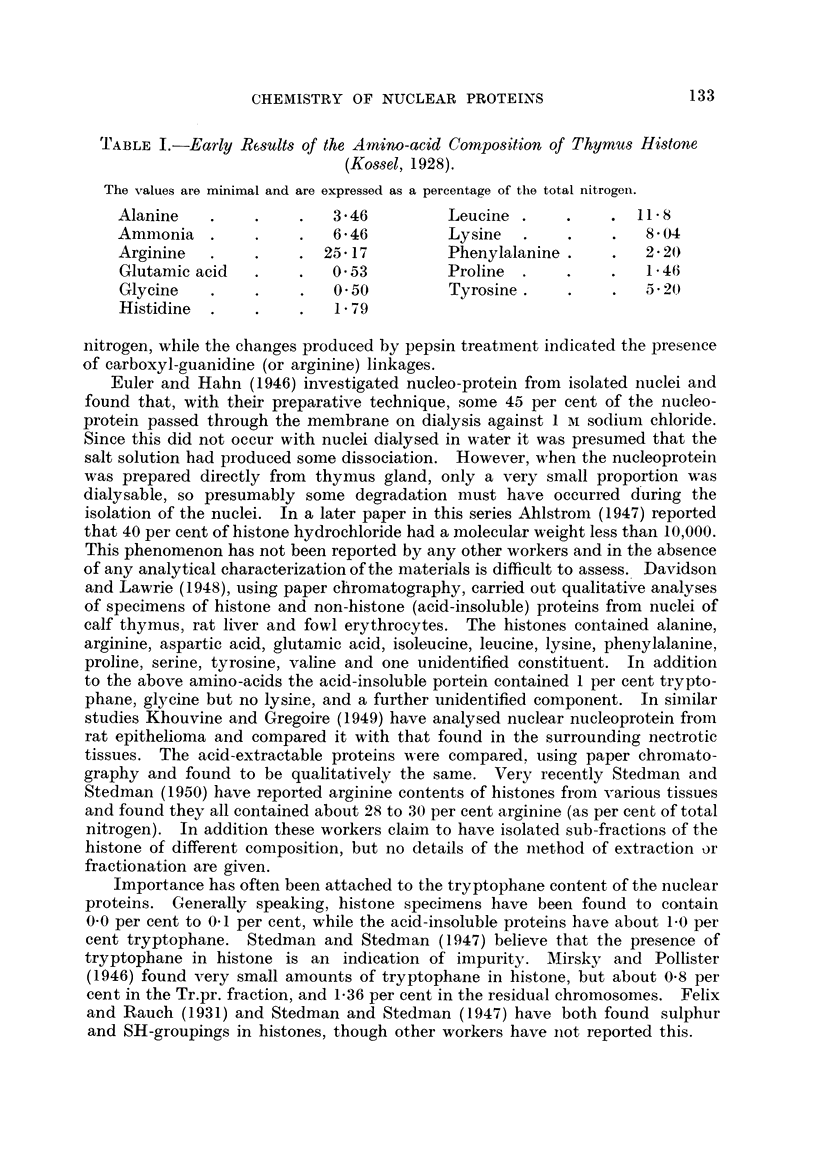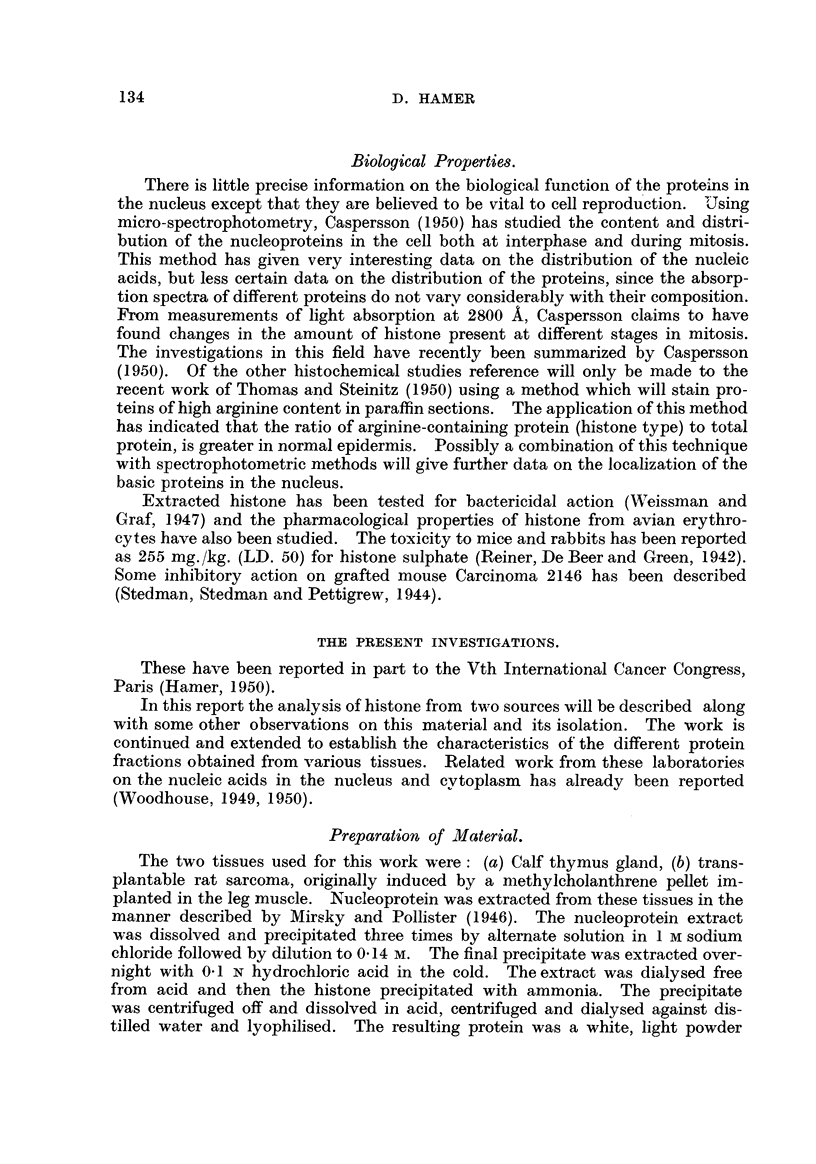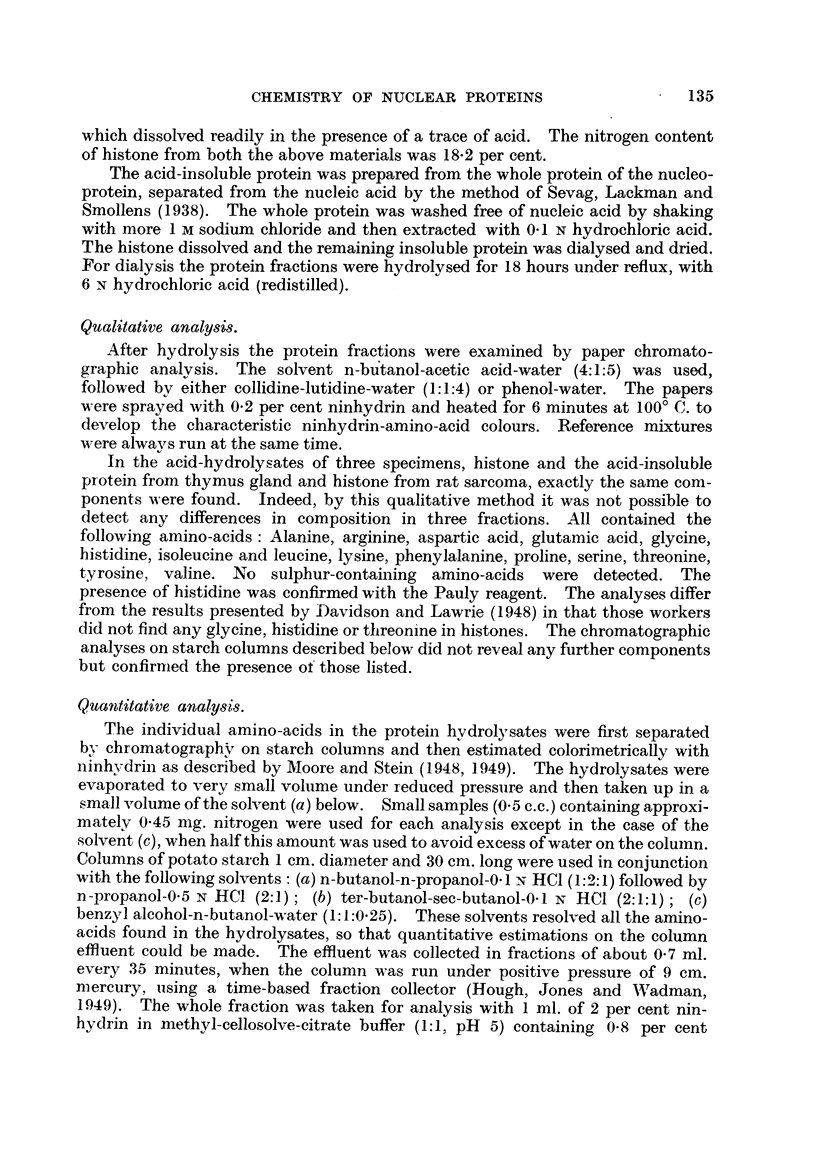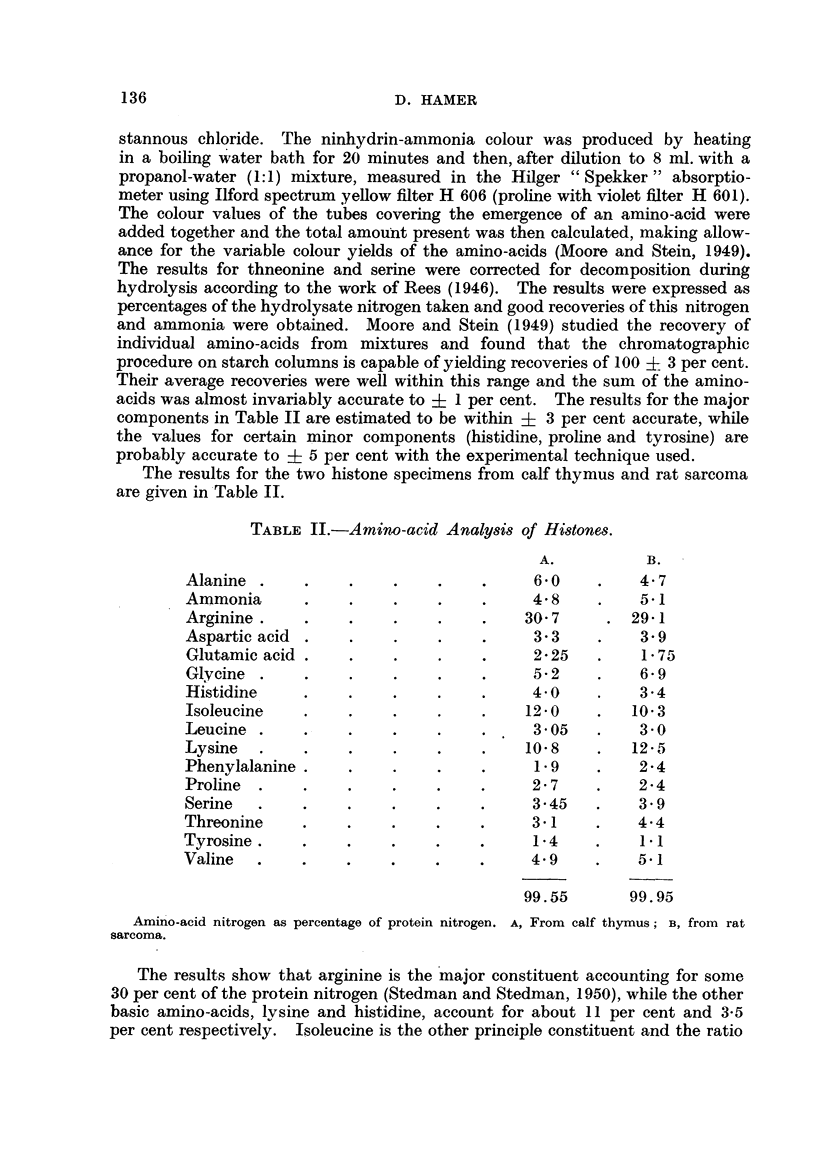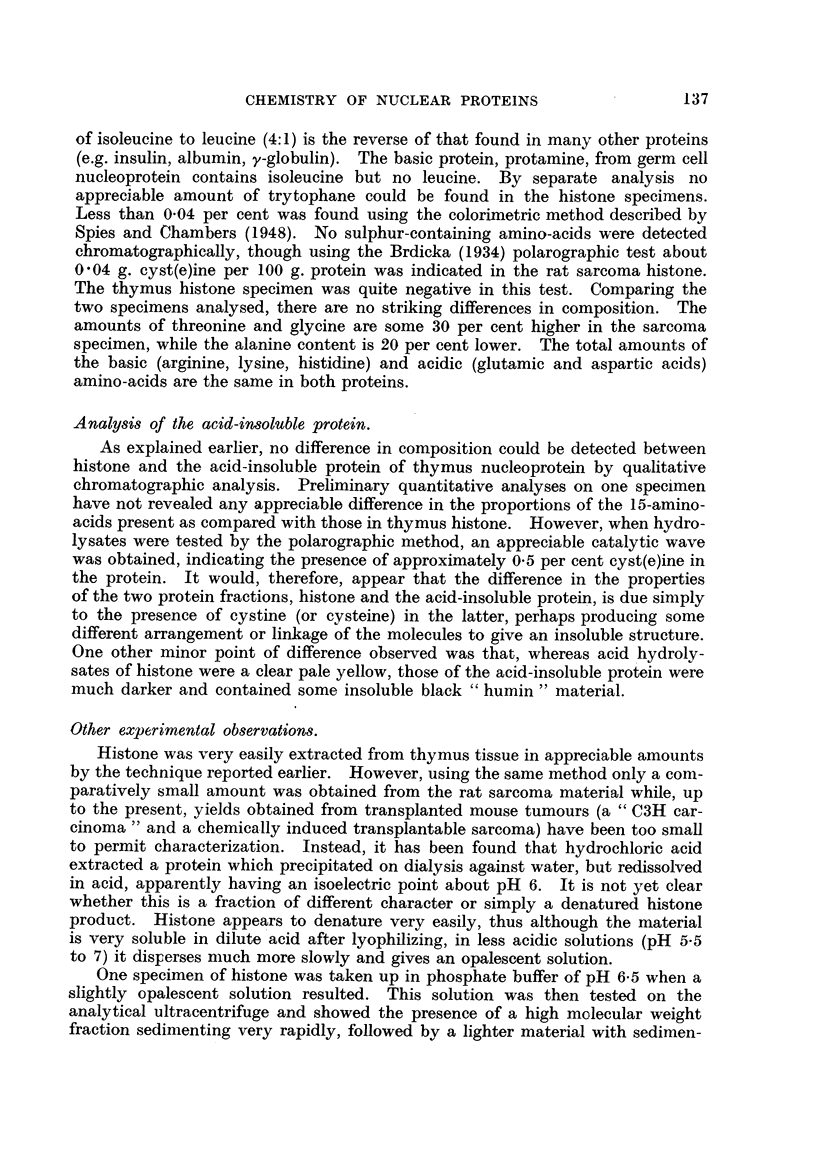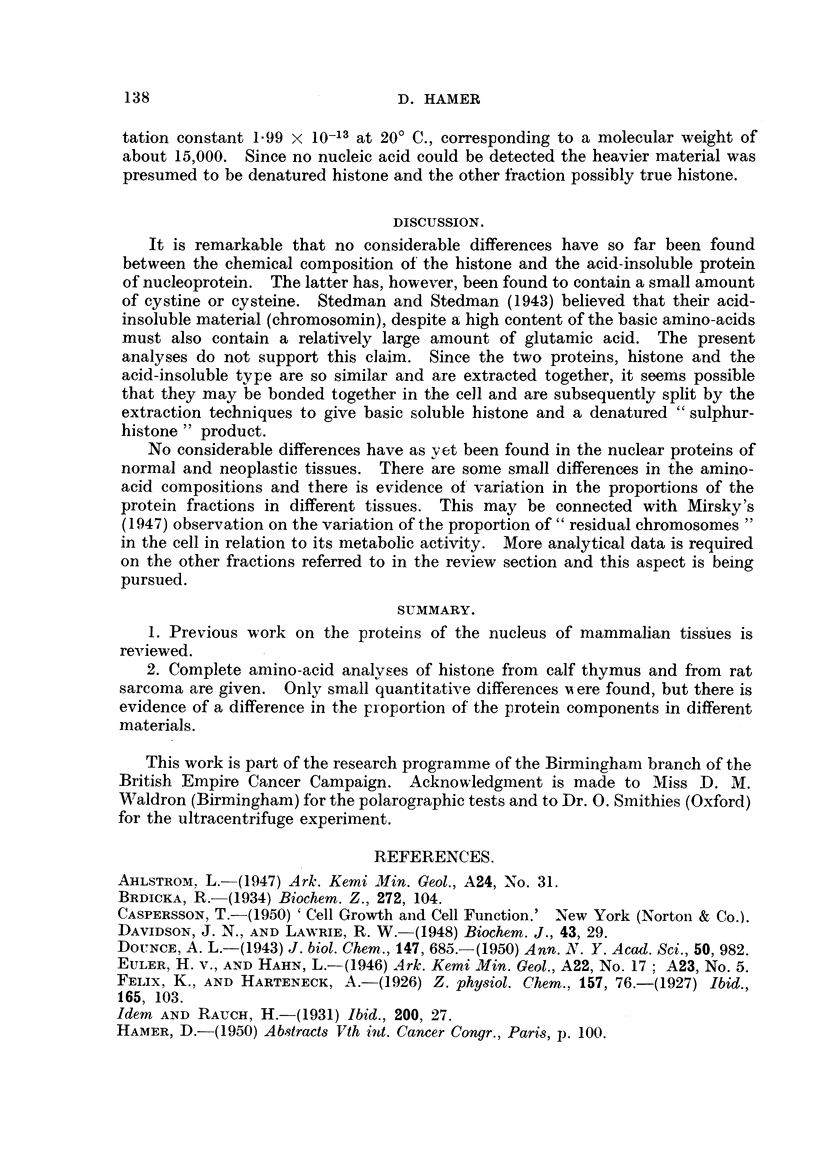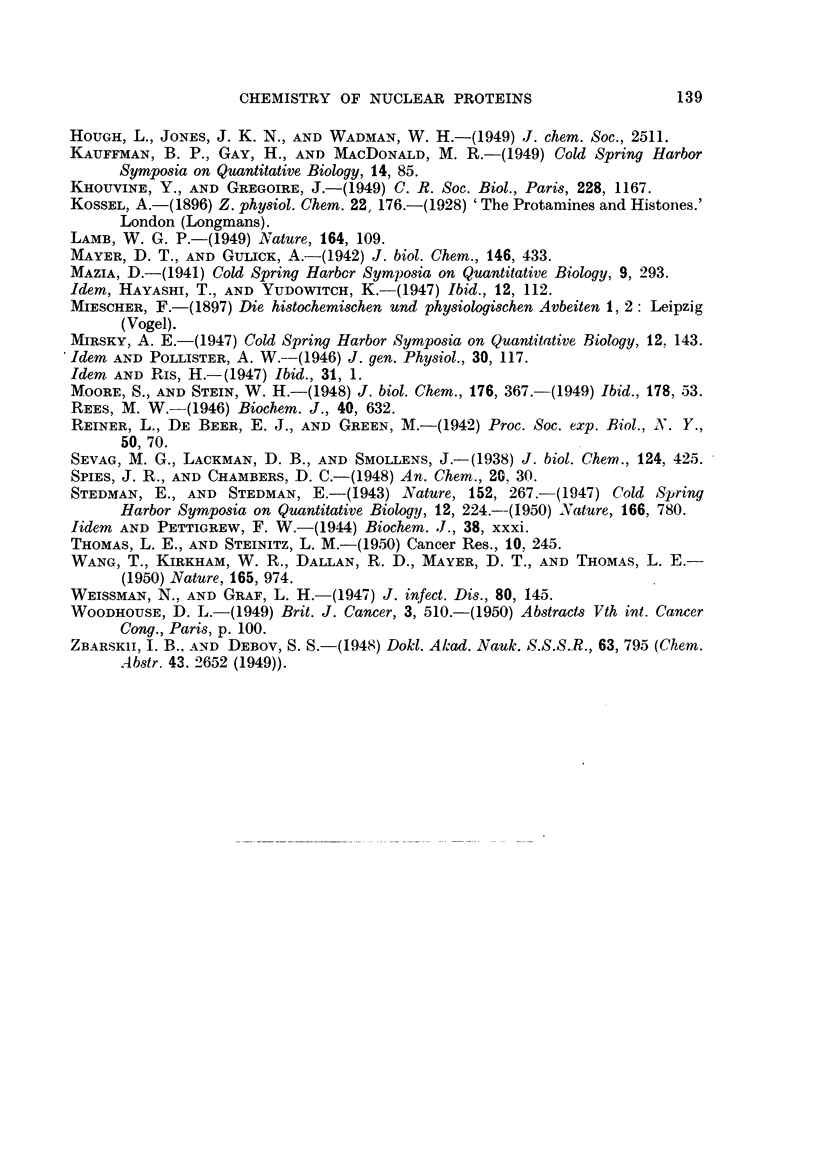# Aspects of the Chemistry of the Proteins in the Nucleus; A Short Review and Some Experimental Results

**DOI:** 10.1038/bjc.1951.13

**Published:** 1951-03

**Authors:** D. Hamer


### ASPECTS OF THE CHEMISTRY OF THE PROTEINS IN THE NUCLEUS ; A SHORT REVIEW AND SOME EXPERIMENTAL RESULTS.

D. HAMER.

*From the Cancer Research Laboratories, Department of Pathology, the Medical School, Edgbaston, Birmingham, 15.*

Received for publication December 29, 1950.

FIFTY years ago various workers, notably Miescher (1897) and Kossel (1896), first extracted material which they termed nucleo-protein and Kossel (1928) carried out investigations on the protein moieties of this substance. In some germ cells (e.g. salmon milt) there appeared to be a simple protein in combination with the nucleic acid which could readily be isolated by virtue of its basic character. Kossel (1928) described similar protein material from various mammalian cells (e.g. calf thymus lymphocytes) which also had basic properties, though a more complicated chemical structure. The two classes of proteins have been termed the protamines and histones, respectively.

These proteins, in association with nucleic acid, i.e. nucleoprotein, are believed to play an important part in cell reproduction, but their basic functions have not been elucidated. Recently developed analytical techniques, particularly chromatographic analysis and the use of the ultra-violet spectrophotometer, have produced considerable fresh data on the composition of the nucleic acids, but much less research has been done on the protein fractions. This paper collects some information on this topic and some analytical results obtained in our laboratory are presented.

### *The Proteins Found in the Nucleus.*

Mirsky and Pollister (1946) were able to dissolve nuclear nucleoprotein in 1 M sodium chloride, after first removing the cytoplasmic material with 0·14 M saline, and to isolate this nucleoprotein by diluting the solution to a salt concentration of 0·14 M, when it precipitates in fibrous form. Similar material could be obtained from isolated nuclei (Dounce, 1943) confirming that these nucleoproteins were of nuclear origin. This extracted material was found to consist of deoxyribonucleic acid and two protein fractions, one of which was classified as a histone, being readily soluble in acid and precipitated out of solution by ammonia. The second protein was characterized by having a higher tryptophane content and being soluble in acid or alkali, and was referred to as the tryptophane-containing protein (Tr.pr.).

Later these observations were extended and it was found that by drastic mechanical treatment of the nuclei followed by differential centrifugation, a material could be obtained consisting of small fibres having certain characteristics similar to chromosomes, when examined under the microscope (Mirsky and Ris, 1947 ; Mirsky, 1947). The conclusions have, however, been questioned by

Lamb (1949), who maintained that under the conditions employed the nuclei are actually torn into fibres, but not necessarily ruptured. Mirsky (1947) found that treatment of the isolated chromosomes with 1 M sodium chloride caused much of the material to pass into solution, as had been observed with the isolated nuclei. Thread-like portions, termed "residual chromosomes," remained undissolved, and contained protein with some nucleic acid. The material extracted was a nucleoprotein containing nucleic acid and histone, but no Tr.pr. It was suggested, therefore, that this must be derived from the residual chromosomes. It must be pointed out, however, that the method which successfully extracted nucleoprotein containing Tr.pr. from isolated nuclei, apparently failed to do so from the chromosomes, and therefore claims respecting the identity of Tr.pr. and the residual chromosomes must await analytical confirmation. It is of special interest that cells which could be considered metabolically active (liver, kidney) contain as much as 50 per cent of the total isolated chromosome material in the form of residual chromosome.

Stedman and Stedman (1943, 1947) also isolated an acid-insoluble protein from the nucleus which they termed "chromosomin," believing that it was the structural protein of the chromosome and that in the nucleus it was embedded in a support of nucleo-histone. The work of both Mirsky (1947) and Stedman and Stedman (1943, 1947) seems to indicate that a protein forms the basic structure of the chromosomes though the part played by the nucleic acid and the histone is uncertain. This is an important aspect but cannot be discussed here. Mention should be made, however, of the work of Mazia (1941) and Mazia, Hyashi and Yudowitch (1947), who found that enzymatically the nucleic acid can be digested away from salivary gland chromosomes without disrupting the structure, but that a proteolytic enzyme would cause disintegration. Kauffmann, Gay and MacDonald (1949) have recently suggested that this work may need further investigation before interpretations on chromosome structure can be made.

Mayer and Gulick (1942) prepared nuclear material by a method in which the tissue is frozen, dried and then fractionated by sedimentation in organic solvents at various specific gravities. From this material they isolated nucleic acid and histone, and in addition a fraction with isoelectric point between 5·8 and 6·15, which constituted an appreciable proportion of the nuclear proteins. The evidence suggested that the fraction contained a sulphur-containing protein with an acidic isoelectric point and a protein of globulin type. If this material was, indeed, of nuclear origin, then this fraction appears to correspond in properties with the "chromosomin" of Stedman and Stedman (1943, 1947). Recently Wang, Kirkham, Dallan, Mayer and Thomas (1950) have reported the isolation of a similar acidic-protein fraction from rat liver nuclei and from calf thymus chromosomes. The nuclei and chromosomes were first extracted with 1 M sodium chloride and then the residue was extracted with dilute sodium hydroxide. Acidification of this extract precipitated a protein which contained about 6 per cent arginine and no nucleic acid. Since the nucleoprotein extracted from the nuclei has been shown to contain nucleic acid histone and Tr.pr., the alkali soluble protein here must be of different character and this is more likely to be the protein of the residual chromosomes than the Tr.pr. fraction. Finally, two Russian workers (Zbarskii and Debov, 1948) have reported that the protein remaining after the extraction of the nucleoprotein consists of two fractions, one of which is weakly acidic in character, being soluble in dilute alkali and having an isoelectric point at pH 5·0 to 5·3 (presumably similar to the fraction reported by Wang, Kirkham, Dallan, Mayer and Thomas (1950)), and secondly a final residue which is insoluble in all the usual extraction media and only swells in alkali.

In the nucleus, therefore, there is evidence of the presence of four protein fractions, in addition to the nucleic acid. Two components are extracted by 1 M sodium chloride in association with the nucleic acid, namely, the histone and an acid-insoluble protein. The protein of the nucleus remaining undissolved after the extraction can then be split into two parts, a fraction soluble in dilute alkali and a remaining insoluble residue (Fig. 1). Further work will be necessary before the role and location of these fractions in the nucleus can be ascertained.

FIG. 1.—The different fractions reported to be present in the nucleus.

It has now been shown that the nucleus has its own specific pattern of enzymes and since these are, presumably, protein in character, they must be considered. Certain enzymes of the liver (e.g. arginase, D-amino-oxidase) have been found to be present in greater concentration in the nucleus than in the whole liver ; alkaline phosphatase is also very abundant, while others, particularly cytochrome oxidase and succinic dehydrogenase, have been found to be much lower. Indeed, with liver cell nuclei, the absence of succinic dehydrogenase activity has been used as a criterion of purity of the preparations. If confirmed, this may possibly have an application to nuclear protein work in proving that only nuclei are being used and so avoiding any cytoplasmic contamination. These aspects have been well reviewed by Dounce (1950).

### *Available Analytical Data.*

Most of the analytical results reported in the literature concern the protein histone ; so far no complete quantitative amino-acid analysis of this or any of the other fractions is available, with the exception of the data of Kossel (1928) summarized in Table I. Arginine was found to be the main constituent, accounting for some 25 per cent of the protein nitrogen, with leucine and lysine next in content.

Felix and Harteneck (1926, 1927) and Felix and Rauch (1931) in a series of papers described the preparation and some analytical investigations on thymus histone. Analyses were reported on histone hydrochloride and a possible minimum molecular weight of 14,200 was estimated. The hydrochloride contained 15·9 per cent nitrogen and 1·87 per cent sulphur, 25·7 per cent of the nitrogen being due to arginine and 5·5 per cent to histidine. In other work it was found that the histone had 20 basic groupings and 7·5 free-COOH groups per 100 atoms

TABLE I.—*Early Results of the Amino-acid Composition of Thymus Histone* (*Kossel*, 1928).

The values are minimal and are expressed as a percentage of the total nitrogen.

| | | | |
|---|---|---|---|
| Alanine | 3·46 | Leucine | 11·8 |
| Ammonia | 6·46 | Lysine | 8·04 |
| Arginine | 25·17 | Phenylalanine | 2·20 |
| Glutamic acid | 0·53 | Proline | 1·46 |
| Glycine | 0·50 | Tyrosine | 5·20 |
| Histidine | 1·79 | | |

nitrogen, while the changes produced by pepsin treatment indicated the presence of carboxyl-guanidine (or arginine) linkages.

Euler and Hahn (1946) investigated nucleo-protein from isolated nuclei and found that, with their preparative technique, some 45 per cent of the nucleoprotein passed through the membrane on dialysis against 1 M sodium chloride. Since this did not occur with nuclei dialysed in water it was presumed that the salt solution had produced some dissociation. However, when the nucleoprotein was prepared directly from thymus gland, only a very small proportion was dialysable, so presumably some degradation must have occurred during the isolation of the nuclei. In a later paper in this series Ahlstrom (1947) reported that 40 per cent of histone hydrochloride had a molecular weight less than 10,000. This phenomenon has not been reported by any other workers and in the absence of any analytical characterization of the materials is difficult to assess. Davidson and Lawrie (1948), using paper chromatography, carried out qualitative analyses of specimens of histone and non-histone (acid-insoluble) proteins from nuclei of calf thymus, rat liver and fowl erythrocytes. The histones contained alanine, arginine, aspartic acid, glutamic acid, isoleucine, leucine, lysine, phenylalanine, proline, serine, tyrosine, valine and one unidentified constituent. In addition to the above amino-acids the acid-insoluble portein contained 1 per cent tryptophane, glycine but no lysine, and a further unidentified component. In similar studies Khouvine and Gregoire (1949) have analysed nuclear nucleoprotein from rat epithelioma and compared it with that found in the surrounding nectrotic tissues. The acid-extractable proteins were compared, using paper chromatography and found to be qualitatively the same. Very recently Stedman and Stedman (1950) have reported arginine contents of histones from various tissues and found they all contained about 28 to 30 per cent arginine (as per cent of total nitrogen). In addition these workers claim to have isolated sub-fractions of the histone of different composition, but no details of the method of extraction or fractionation are given.

Importance has often been attached to the tryptophane content of the nuclear proteins. Generally speaking, histone specimens have been found to contain 0·0 per cent to 0·1 per cent, while the acid-insoluble proteins have about 1·0 per cent tryptophane. Stedman and Stedman (1947) believe that the presence of tryptophane in histone is an indication of impurity. Mirsky and Pollister (1946) found very small amounts of tryptophane in histone, but about 0·8 per cent in the Tr.pr. fraction, and 1·36 per cent in the residual chromosomes. Felix and Rauch (1931) and Stedman and Stedman (1947) have both found sulphur and SH-groupings in histones, though other workers have not reported this.

*Biological Properties.*

There is little precise information on the biological function of the proteins in the nucleus except that they are believed to be vital to cell reproduction. Using micro-spectrophotometry, Caspersson (1950) has studied the content and distribution of the nucleoproteins in the cell both at interphase and during mitosis. This method has given very interesting data on the distribution of the nucleic acids, but less certain data on the distribution of the proteins, since the absorption spectra of different proteins do not vary considerably with their composition. From measurements of light absorption at 2800 Å, Caspersson claims to have found changes in the amount of histone present at different stages in mitosis. The investigations in this field have recently been summarized by Caspersson (1950). Of the other histochemical studies reference will only be made to the recent work of Thomas and Steinitz (1950) using a method which will stain proteins of high arginine content in paraffin sections. The application of this method has indicated that the ratio of arginine-containing protein (histone type) to total protein, is greater in normal epidermis. Possibly a combination of this technique with spectrophotometric methods will give further data on the localization of the basic proteins in the nucleus.

Extracted histone has been tested for bactericidal action (Weissman and Graf, 1947) and the pharmacological properties of histone from avian erythrocytes have also been studied. The toxicity to mice and rabbits has been reported as 255 mg./kg. (LD. 50) for histone sulphate (Reiner, De Beer and Green, 1942). Some inhibitory action on grafted mouse Carcinoma 2146 has been described (Stedman, Stedman and Pettigrew, 1944).

### THE PRESENT INVESTIGATIONS.

These have been reported in part to the Vth International Cancer Congress, Paris (Hamer, 1950).

In this report the analysis of histone from two sources will be described along with some other observations on this material and its isolation. The work is continued and extended to establish the characteristics of the different protein fractions obtained from various tissues. Related work from these laboratories on the nucleic acids in the nucleus and cytoplasm has already been reported (Woodhouse, 1949, 1950).

*Preparation of Material.*

The two tissues used for this work were : (*a*) Calf thymus gland, (*b*) transplantable rat sarcoma, originally induced by a methylcholanthrene pellet implanted in the leg muscle. Nucleoprotein was extracted from these tissues in the manner described by Mirsky and Pollister (1946). The nucleoprotein extract was dissolved and precipitated three times by alternate solution in 1 M sodium chloride followed by dilution to 0·14 M. The final precipitate was extracted overnight with 0·1 N hydrochloric acid in the cold. The extract was dialysed free from acid and then the histone precipitated with ammonia. The precipitate was centrifuged off and dissolved in acid, centrifuged and dialysed against distilled water and lyophilised. The resulting protein was a white, light powder which dissolved readily in the presence of a trace of acid. The nitrogen content of histone from both the above materials was 18·2 per cent.

The acid-insoluble protein was prepared from the whole protein of the nucleoprotein, separated from the nucleic acid by the method of Sevag, Lackman and Smollens (1938). The whole protein was washed free of nucleic acid by shaking with more 1 M sodium chloride and then extracted with 0·1 N hydrochloric acid. The histone dissolved and the remaining insoluble protein was dialysed and dried. For dialysis the protein fractions were hydrolysed for 18 hours under reflux, with 6 N hydrochloric acid (redistilled).

*Qualitative analysis.*

After hydrolysis the protein fractions were examined by paper chromatographic analysis. The solvent n-butanol-acetic acid-water (4:1:5) was used, followed by either collidine-lutidine-water (1:1:4) or phenol-water. The papers were sprayed with 0·2 per cent ninhydrin and heated for 6 minutes at 100° C. to develop the characteristic ninhydrin-amino-acid colours. Reference mixtures were always run at the same time.

In the acid-hydrolysates of three specimens, histone and the acid-insoluble protein from thymus gland and histone from rat sarcoma, exactly the same components were found. Indeed, by this qualitative method it was not possible to detect any differences in composition in three fractions. All contained the following amino-acids : Alanine, arginine, aspartic acid, glutamic acid, glycine, histidine, isoleucine and leucine, lysine, phenylalanine, proline, serine, threonine, tyrosine, valine. No sulphur-containing amino-acids were detected. The presence of histidine was confirmed with the Pauly reagent. The analyses differ from the results presented by Davidson and Lawrie (1948) in that those workers did not find any glycine, histidine or threonine in histones. The chromatographic analyses on starch columns described below did not reveal any further components but confirmed the presence of those listed.

*Quantitative analysis.*

The individual amino-acids in the protein hydrolysates were first separated by chromatography on starch columns and then estimated colorimetrically with ninhydrin as described by Moore and Stein (1948, 1949). The hydrolysates were evaporated to very small volume under reduced pressure and then taken up in a small volume of the solvent (*a*) below. Small samples (0·5 c.c.) containing approximately 0·45 mg. nitrogen were used for each analysis except in the case of the solvent (*c*), when half this amount was used to avoid excess of water on the column. Columns of potato starch 1 cm. diameter and 30 cm. long were used in conjunction with the following solvents : (*a*) n-butanol-n-propanol-0·1 N HCl (1:2:1) followed by n-propanol-0·5 N HCl (2:1) ; (*b*) ter-butanol-sec-butanol-0·1 N HCl (2:1:1) ; (*c*) benzyl alcohol-n-butanol-water (1:1:0·25). These solvents resolved all the amino-acids found in the hydrolysates, so that quantitative estimations on the column effluent could be made. The effluent was collected in fractions of about 0·7 ml. every 35 minutes, when the column was run under positive pressure of 9 cm. mercury, using a time-based fraction collector (Hough, Jones and Wadman, 1949). The whole fraction was taken for analysis with 1 ml. of 2 per cent ninhydrin in methyl-cellosolve-citrate buffer (1:1, pH 5) containing 0·8 per cent stannous chloride. The ninhydrin-ammonia colour was produced by heating in a boiling water bath for 20 minutes and then, after dilution to 8 ml. with a propanol-water (1:1) mixture, measured in the Hilger "Spekker" absorptiometer using Ilford spectrum yellow filter H 606 (proline with violet filter H 601). The colour values of the tubes covering the emergence of an amino-acid were added together and the total amount present was then calculated, making allowance for the variable colour yields of the amino-acids (Moore and Stein, 1949). The results for thneonine and serine were corrected for decomposition during hydrolysis according to the work of Rees (1946). The results were expressed as percentages of the hydrolysate nitrogen taken and good recoveries of this nitrogen and ammonia were obtained. Moore and Stein (1949) studied the recovery of individual amino-acids from mixtures and found that the chromatographic procedure on starch columns is capable of yielding recoveries of 100 ± 3 per cent. Their average recoveries were well within this range and the sum of the amino-acids was almost invariably accurate to ± 1 per cent. The results for the major components in Table II are estimated to be within ± 3 per cent accurate, while the values for certain minor components (histidine, proline and tyrosine) are probably accurate to ± 5 per cent with the experimental technique used.

The results for the two histone specimens from calf thymus and rat sarcoma are given in Table II.

TABLE II.—*Amino-acid Analysis of Histones.*

| | A. | B. |
|---|---|---|
| Alanine | 6·0 | 4·7 |
| Ammonia | 4·8 | 5·1 |
| Arginine | 30·7 | 29·1 |
| Aspartic acid | 3·3 | 3·9 |
| Glutamic acid | 2·25 | 1·75 |
| Glycine | 5·2 | 6·9 |
| Histidine | 4·0 | 3·4 |
| Isoleucine | 12·0 | 10·3 |
| Leucine | 3·05 | 3·0 |
| Lysine | 10·8 | 12·5 |
| Phenylalanine | 1·9 | 2·4 |
| Proline | 2·7 | 2·4 |
| Serine | 3·45 | 3·9 |
| Threonine | 3·1 | 4·4 |
| Tyrosine | 1·4 | 1·1 |
| Valine | 4·9 | 5·1 |
| | 99.55 | 99.95 |

Amino-acid nitrogen as percentage of protein nitrogen. A, From calf thymus; B, from rat sarcoma.

The results show that arginine is the major constituent accounting for some 30 per cent of the protein nitrogen (Stedman and Stedman, 1950), while the other basic amino-acids, lysine and histidine, account for about 11 per cent and 3·5 per cent respectively. Isoleucine is the other principle constituent and the ratio of isoleucine to leucine (4:1) is the reverse of that found in many other proteins (e.g. insulin, albumin, γ-globulin). The basic protein, protamine, from germ cell nucleoprotein contains isoleucine but no leucine. By separate analysis no appreciable amount of trytophane could be found in the histone specimens. Less than 0·04 per cent was found using the colorimetric method described by Spies and Chambers (1948). No sulphur-containing amino-acids were detected chromatographically, though using the Brdicka (1934) polarographic test about 0·04 g. cyst(e)ine per 100 g. protein was indicated in the rat sarcoma histone. The thymus histone specimen was quite negative in this test. Comparing the two specimens analysed, there are no striking differences in composition. The amounts of threonine and glycine are some 30 per cent higher in the sarcoma specimen, while the alanine content is 20 per cent lower. The total amounts of the basic (arginine, lysine, histidine) and acidic (glutamic and aspartic acids) amino-acids are the same in both proteins.

*Analysis of the acid-insoluble protein.*

As explained earlier, no difference in composition could be detected between histone and the acid-insoluble protein of thymus nucleoprotein by qualitative chromatographic analysis. Preliminary quantitative analyses on one specimen have not revealed any appreciable difference in the proportions of the 15-amino-acids present as compared with those in thymus histone. However, when hydrolysates were tested by the polarographic method, an appreciable catalytic wave was obtained, indicating the presence of approximately 0·5 per cent cyst(e)ine in the protein. It would, therefore, appear that the difference in the properties of the two protein fractions, histone and the acid-insoluble protein, is due simply to the presence of cystine (or cysteine) in the latter, perhaps producing some different arrangement or linkage of the molecules to give an insoluble structure. One other minor point of difference observed was that, whereas acid hydrolysates of histone were a clear pale yellow, those of the acid-insoluble protein were much darker and contained some insoluble black " humin " material.

*Other experimental observations.*

Histone was very easily extracted from thymus tissue in appreciable amounts by the technique reported earlier. However, using the same method only a comparatively small amount was obtained from the rat sarcoma material while, up to the present, yields obtained from transplanted mouse tumours (a " C3H carcinoma " and a chemically induced transplantable sarcoma) have been too small to permit characterization. Instead, it has been found that hydrochloric acid extracted a protein which precipitated on dialysis against water, but redissolved in acid, apparently having an isoelectric point about pH 6. It is not yet clear whether this is a fraction of different character or simply a denatured histone product. Histone appears to denature very easily, thus although the material is very soluble in dilute acid after lyophilizing, in less acidic solutions (pH 5·5 to 7) it disperses much more slowly and gives an opalescent solution.

One specimen of histone was taken up in phosphate buffer of pH 6·5 when a slightly opalescent solution resulted. This solution was then tested on the analytical ultracentrifuge and showed the presence of a high molecular weight fraction sedimenting very rapidly, followed by a lighter material with sedimentation constant $1 \cdot 99 \times 10^{-13}$ at 20° C., corresponding to a molecular weight of about 15,000. Since no nucleic acid could be detected the heavier material was presumed to be denatured histone and the other fraction possibly true histone.

### DISCUSSION.

It is remarkable that no considerable differences have so far been found between the chemical composition of the histone and the acid-insoluble protein of nucleoprotein. The latter has, however, been found to contain a small amount of cystine or cysteine. Stedman and Stedman (1943) believed that their acid-insoluble material (chromosomin), despite a high content of the basic amino-acids must also contain a relatively large amount of glutamic acid. The present analyses do not support this claim. Since the two proteins, histone and the acid-insoluble type are so similar and are extracted together, it seems possible that they may be bonded together in the cell and are subsequently split by the extraction techniques to give basic soluble histone and a denatured " sulphur-histone " product.

No considerable differences have as yet been found in the nuclear proteins of normal and neoplastic tissues. There are some small differences in the amino-acid compositions and there is evidence of variation in the proportions of the protein fractions in different tissues. This may be connected with Mirsky's (1947) observation on the variation of the proportion of " residual chromosomes " in the cell in relation to its metabolic activity. More analytical data is required on the other fractions referred to in the review section and this aspect is being pursued.

### SUMMARY.

1. Previous work on the proteins of the nucleus of mammalian tissues is reviewed.

2. Complete amino-acid analyses of histone from calf thymus and from rat sarcoma are given. Only small quantitative differences were found, but there is evidence of a difference in the proportion of the protein components in different materials.

This work is part of the research programme of the Birmingham branch of the British Empire Cancer Campaign. Acknowledgment is made to Miss D. M. Waldron (Birmingham) for the polarographic tests and to Dr. O. Smithies (Oxford) for the ultracentrifuge experiment.

### REFERENCES.

AHLSTROM, L.—(1947) *Ark. Kemi Min. Geol.*, A**24**, No. 31.
BRDICKA, R.—(1934) *Biochem. Z.*, **272**, 104.
CASPERSSON, T.—(1950) ' Cell Growth and Cell Function.' New York (Norton & Co.).
DAVIDSON, J. N., AND LAWRIE, R. W.—(1948) *Biochem. J.*, **43**, 29.
DOUNCE, A. L.—(1943) *J. biol. Chem.*, **147**, 685.—(1950) *Ann. N. Y. Acad. Sci.*, **50**, 982.
EULER, H. v., AND HAHN, L.—(1946) *Ark. Kemi Min. Geol.*, A**22**, No. 17 ; A**23**, No. 5.
FELIX, K., AND HARTENECK, A.—(1926) *Z. physiol. Chem.*, **157**, 76.—(1927) *Ibid.*, **165**, 103.
*Idem* AND RAUCH, H.—(1931) *Ibid.*, **200**, 27.
HAMER, D.—(1950) *Abstracts Vth int. Cancer Congr., Paris*, p. 100.

HOUGH, L., JONES, J. K. N., AND WADMAN, W. H.—(1949) *J. chem. Soc.*, 2511.
KAUFFMAN, B. P., GAY, H., AND MACDONALD, M. R.—(1949) *Cold Spring Harbor Symposia on Quantitative Biology*, **14**, 85.
KHOUVINE, Y., AND GREGOIRE, J.—(1949) *C. R. Soc. Biol., Paris*, **228**, 1167.
KOSSEL, A.—(1896) *Z. physiol. Chem.* **22**, 176.—(1928) 'The Protamines and Histones.' London (Longmans).
LAMB, W. G. P.—(1949) *Nature*, **164**, 109.
MAYER, D. T., AND GULICK, A.—(1942) *J. biol. Chem.*, **146**, 433.
MAZIA, D.—(1941) *Cold Spring Harbcr Symposia on Quantitative Biology*, **9**, 293.
*Idem*, HAYASHI, T., AND YUDOWITCH, K.—(1947) *Ibid.*, **12**, 112.
MIESCHER, F.—(1897) *Die histochemischen und physiologischen Avbeiten* **1**, 2 : Leipzig (Vogel).
MIRSKY, A. E.—(1947) *Cold Spring Harbor Symposia on Quantitative Biology*, **12**, 143.
*Idem* AND POLLISTER, A. W.—(1946) *J. gen. Physiol.*, **30**, 117.
*Idem* AND RIS, H.—(1947) *Ibid.*, **31**, 1.
MOORE, S., AND STEIN, W. H.—(1948) *J. biol. Chem.*, **176**, 367.—(1949) *Ibid.*, **178**, 53.
REES, M. W.—(1946) *Biochem. J.*, **40**, 632.
REINER, L., DE BEER, E. J., AND GREEN, M.—(1942) *Proc. Soc. exp. Biol., N. Y.*, **50**, 70.
SEVAG, M. G., LACKMAN, D. B., AND SMOLLENS, J.—(1938) *J. biol. Chem.*, **124**, 425.
SPIES, J. R., AND CHAMBERS, D. C.—(1948) *An. Chem.*, **20**, 30.
STEDMAN, E., AND STEDMAN, E.—(1943) *Nature*, **152**, 267.—(1947) *Cold Spring Harbor Symposia on Quantitative Biology*, **12**, 224.—(1950) *Nature*, **166**, 780.
*Iidem* AND PETTIGREW, F. W.—(1944) *Biochem. J.*, **38**, xxxi.
THOMAS, L. E., AND STEINITZ, L. M.—(1950) Cancer Res., **10**, 245.
WANG, T., KIRKHAM, W. R., DALLAN, R. D., MAYER, D. T., AND THOMAS, L. E.—(1950) *Nature*, **165**, 974.
WEISSMAN, N., AND GRAF, L. H.—(1947) *J. infect. Dis.*, **80**, 145.
WOODHOUSE, D. L.—(1949) *Brit. J. Cancer*, **3**, 510.—(1950) *Abstracts Vth int. Cancer Cong., Paris*, p. 100.
ZBARSKII, I. B., AND DEBOV, S. S.—(1948) *Dokl. Akad. Nauk. S.S.S.R.*, **63**, 795 (*Chem. Abstr.* **43**, 2652 (1949)).